# Study on the Design and Performance of a Glove Based on the FBG Array for Hand Posture Sensing

**DOI:** 10.3390/s23208495

**Published:** 2023-10-16

**Authors:** Hongcheng Rao, Binbin Luo, Decao Wu, Pan Yi, Fudan Chen, Shenghui Shi, Xue Zou, Yuliang Chen, Mingfu Zhao

**Affiliations:** Chongqing Key Laboratory of Optical Fiber Sensor and Photoelectric Detection, Chongqing University of Technology, Chongqing 400054, China; 563296885@cqut.edu.cn (H.R.); wudecao@cqut.edu.cn (D.W.); yipan9710@163.com (P.Y.); 51220710119@cqut.edu.cn (F.C.); shshill@vip.cqut.edu.cn (S.S.); zouxue@cqut.edu.cn (X.Z.); 15172971650@163.com (Y.C.); zmf@cqut.edu.cn (M.Z.)

**Keywords:** fiber Bragg gratings, glove, bending, hand posture perception, object prediction

## Abstract

This study introduces a new wearable fiber-optic sensor glove. The glove utilizes a flexible material, polydimethylsiloxane (PDMS), and a silicone tube to encapsulate fiber Bragg gratings (FBGs). It is employed to enable the self-perception of hand posture, gesture recognition, and the prediction of grasping objects. The investigation employs the Support Vector Machine (SVM) approach for predicting grasping objects. The proposed fiber-optic sensor glove can concurrently monitor the motion of 14 hand joints comprising 5 metacarpophalangeal joints (MCP), 5 proximal interphalangeal joints (PIP), and 4 distal interphalangeal joints (DIP). To expand the measurement range of the sensors, a sinusoidal layout incorporates the FBG array into the glove. The experimental results indicate that the wearable sensing glove can track finger flexion within a range of 0° to 100°, with a modest minimum measurement error (Error) of 0.176° and a minimum standard deviation (SD) of 0.685°. Notably, the glove accurately detects hand gestures in real-time and even forecasts grasping actions. The fiber-optic smart glove technology proposed herein holds promising potential for industrial applications, including object grasping, 3D displays via virtual reality, and human–computer interaction.

## 1. Introduction

In advanced mechatronic systems, robots and humans collaborate in industrial scenarios. Robots undertake high-risk and high-intensity tasks to improve productivity and safety, while humans handle intricate tasks, utilizing their intelligence and creativity to ensure efficient operations and production. In this context, the real-time detection and display of hand movements have become integral technologies and are extensively used in underwater exploration, medical treatment, robotic surgical treatment, and rehabilitation. By assessing hand movements in real-time, valuable information on hand posture and trajectory can be gathered, enabling us to comprehend human movement mechanisms, evaluate patient rehabilitation progress, and operate industrial remote-controlled robots effectively [1,2,3]. Despite the advancements in research on human–robot interaction, achieving natural interaction is still a challenge due to the complexity and variability of human gestures [4,5,6,7]. Fiber grating sensing technology presents a uniquely advantageous solution as it integrates optical signal transmission and sensing functions into a single optical fiber [8], reducing the number and size of the electrical devices needed for sensors. This enhances the compactness, lightweight characteristics, and wide device integration of fiber gratings. The object monitoring of strain or temperature change in the fiber enables the highly sensitive measurement of an object’s shape or temperature [9]. Therefore, the real-time detection and accurate capture of even minor changes in objects permit precise human–computer interaction [10]. Additionally, fiber gratings possess the capability to be multiplexed, enabling the tracking and measurement of numerous locations simultaneously by producing various fiber gratings in an optical fiber [11]. As a result, they exhibit exceptional functioning in multi-user, multi-task HCI environments [12]. Of utmost importance, traditional non-contact sensors are prone to interference from fluctuations in ambient temperature and contamination, which can cause a decrease in performance [13]. In contrast, fiber-optic grating sensors have excellent immunity to electromagnetic interference, which improves the system’s reliability and stability [14]. Moreover, they are not affected by ambient line-of-sight occlusion.

In the study of fiber-optic gratings in human–computer interaction, many scholars have attained significant progress and accomplishments. For instance, GUO et al. [15] engineered a wearable sensor utilizing stretchable fiber Bragg grating (FBG) technology, which could measure up to 50% of the dynamic strain in tension, bending, and torsion. Chandan et al. [16] crafted medical rehabilitation sensor gloves that incorporated FBG technology to achieve an angular resolution of 0.1° and a measurement range of 80°. Pasqual et al. [17] employed strain measurements to estimate the curvature of the measured structure, achieving a curvature sensitivity of 1.39 nm/m^−1^ and a resolution of 7.2 × 10^−4^. Cui et al. [18] suggested a reconstruction method for 3D spatial curves with an error of merely 17.9%. Furthermore, Sun et al. [19] designed a technique to monitor the shape of the envelope of a close airship, with a shape error of 4.82%. In addition, Li et al. [20] developed gloves for measuring joint angles with high sensitivity (5.6 pm/°), which had the ability to differentiate between different joint movements. In these studies, selecting the right encapsulation material is fundamental to the gloves’ performance. While inflexible materials such as polyimide and polyvinyl chloride [21] have been widely used in previous studies, flexible and malleable materials are better suited to guarantee that the sensors adapt to different joint motions and deformations [22]. The properties of pliable materials entailed greater versatility and comfort, facilitating the integration of the sensor within its application environment to obtain more precise measurements. By contrast, inflexible encapsulation materials were superior in terms of structural stability and external environmental protection, safeguarding the sensors against external interferences and increments in damages. Therefore, there existed a compromise between adaptability and inflexibility when deciding on packaging materials for the satisfaction of angle sensors’ requirements in regard to steadiness, responsiveness, and real-time capability.

In this work, a wearable hand posture sensor based on FBG arrays is proposed and experimentally validated. First, the sensor package structure is fabricated by encapsulating the FBG array in a PDMS and silicone tube, and then is integrated into the wearable glove through a sinusoidal arrangement. A LabVIEW-based fiber grating demodulator host system is designed to demodulate and reconstruct the position information of the FBG wavelength variations. By fusing the angular information of multiple FBG measurement points and using a regression model, the 3D shape coordinates of each measurement point in space can be obtained to achieve real-time attitude monitoring. The study also discusses the sensing range, sensing limits, sensing sensitivity, repeatability, and accuracy of gesture recognition and object grasping. Compared to previous studies, this work has innovated the package structure to improve the sensitivity and stability of the sensing unit. In addition, we have successfully advanced pure measurements on joint motion as well as motion pattern discrimination for its application in the field of virtual reality. Finally, our research has important applications in the industrial field by allowing the prediction of object types through different gesture classifications in cases where the visual recognition of objects is not possible. We believe that these new innovative findings are important for the further development of the field. The proposed FBG-based glove has promising applications in industrial human–computer interaction (HCI) due to its ease of manufacture, large measurement range, and high repeatability.

## 2. Principle and Packaging

### 2.1. Materials and Instruments

FBGs are written inside single-mode fibers with a core diameter of 8 µm, cladding diameter of 125 µm, grating length of 10 mm, and 3 dB bandwidth of approximately 0.25 nm. Sylgard Dow Corning PDMS184 is used as the encapsulation material.

An optical fiber grating demodulator (GC-97001C-02-02-A-F, wavelength range: 1525–1565 nm, 1 kHz, accuracy: 2 pm) is used to demodulate the central wavelength variation of the FBGs. For optical fiber fusion splicing, a fusion splicer for fiber-optic cables (Fujimura, TYPE-81C, Japan) and a fiber-optic cable cutter (Fujimura, CT-38, Japan) are necessary. An electric blast dryer (GZX-9000 MBE) from Shanghai Boxun Industrial Co., Ltd., Shanghai, China. is used to encapsulate and cure the sensors. We use an inertial sensor unit (CMP10A) from AWE Intelligent Technology to measure the reference angle value in real-time, which includes a 3-axis gyroscope, 3-axis accelerometer, and 3-axis magnetometer and barometer, with an SD of 0.1° in the attitude measurement error.

### 2.2. Principle of FBG

FBG is a device designed to produce refractive index changes using the photosensitive properties of optical fibers. This passive device operates by exposing the photosensitive fiber to a UV beam that interferes with the incident light, creating light wave fringes that result in a refractive index modulation distribution within the fiber. The resonance equation for the FBG is provided by [22].

(1) $$\lambda_{b}=2n_{\mathrm{eff}}\Lambda$$

where $\lambda_{b}$ represents the central wavelength of the FBG, $n_{\mathrm{eff}}$ denotes the effective refractive index of the fiber, and Λ signifies the grating period.

The FBG’s functionality is impacted by axial strain and temperature, which cause a wavelength drift in the fiber grating. In order to determine this drift, one cannot overlook the influence of the thermal expansion coefficient, elasticity coefficient, and thermo-optic coefficient of the fiber material. These factors, as described in the literature [22], come together to affect the properties and functionality of the fiber grating.

(2) $$\frac{\Delta \lambda_{b}}{\lambda_{b}}=(\alpha_{f}+\xi )\Delta T+(1-P_{e})\Delta \varepsilon$$

where the wavelength drifts by $\Delta \lambda_{b}$, the center wavelength at the start is $\lambda_{b}$, the fiber’s thermal expansion coefficient is $\alpha_{f}$, the thermo-optic coefficient is ξ, and $P_{e}$ equals 0.22 at standard room temperature.

### 2.3. Simulation and Packaging

**Figure 1:**
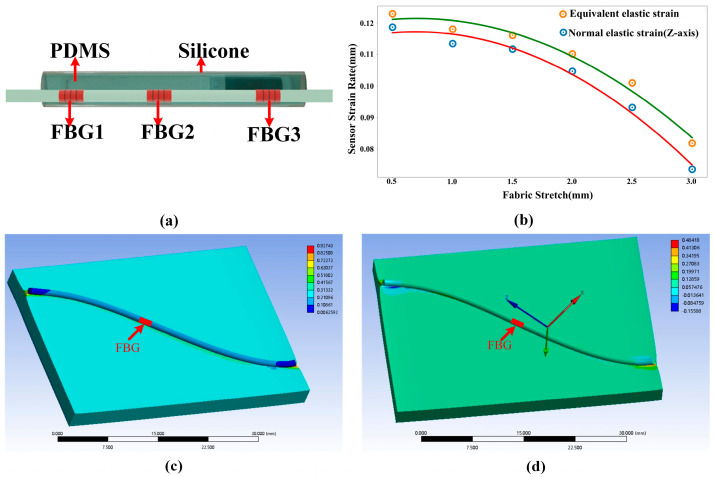
(**a**) Sensor package structure, (**b**) strain response versus package thickness, (**c**) over-all elastic strain simulation (1 mm), (**d**) response of axial (*Z*-axis) elastic strain (1 mm).

The structure of the attitude sensor’s packaging can be observed in Figure 1a. First, a serial array of three FBGs is eccentrically inserted into a silicone tube. A PDMS precursor is formulated by combining hardener and PDMS in a ratio of 1:10. The PDMS precursor is injected directly into the silicone tube using a syringe. Finally, the sensor precursor is placed in a drying oven at a temperature setting of 100 degrees Celsius and cured for 90 min to form the FBG serial attitude sensor unit.

On this basis, we utilize the ANSYS(2021R2) simulation software to simulate and analyze the encapsulated structure. During the simulation, the thickness of the textile is established at 3 mm (density 601.35 kg/m^−3^, Young’s modulus 0.42 MPa, Poisson’s ratio 0.26). The encapsulated structure includes the Material Constant C10 of 0.24324 MPa, the Material Constant C01 of 0.060811 MPa, and the Incompressibility Parameter D1 of 0.13333 MPa^−1^. The contact method between the two is set to bound contact, hexahedral meshing is performed, and the tensile length is set at 9 mm to simulate the strain produced at the FBG position upon glove stretching. Based on the results of the analysis depicted in Figure 1b, it is discovered that the sensor strain (both total and axial strain), for both silicone tubing and PDMS encapsulation, diminishes as the diameter of the silicone tubing increases. Nonetheless, it is also noted that thicker sensor structures offer superior physical protection, enabling them to tolerate the influence of external temperature and pressure more effectively. After considering both strain and physical protection, we conclude that the best option is to enclose the 1 mm outer diameter and 0.5 mm inner diameter PDMS filled with silicone. Figure 1c,d exhibit the overall and axial strains of this encompassed arrangement.

**Figure 2:**
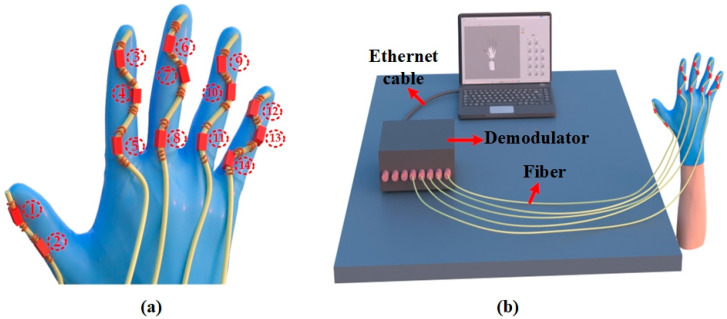
(**a**) Schematic diagram of sensor integration (serial number is FBGs’ number), (**b**) attitude reconfiguration system.

As illustrated in Figure 2a, a glove is fitted with five serialized sensing arrays containing FBG sensors (serial number is the FBGs’ number). The arrays are sewn onto the glove to monitor the MCP, PIP, and DIP joints of the hand. The thumb is monitored with two FBG units, while the remaining fingers are monitored with a total of twelve FBG sensors. The sensors collect data on the center wavelength based on the motion of the joints. To expand the sensor units’ measurement range, we employ a sinusoidal design (with a curvature of around 0.1 mm^−1^ at the bend) for unencumbered joint rotation. It is important to mention that pre-bending the FBG sensor unit alters its grating period and causes a red-drift of about 0.1 nm in its Bragg wavelength at a curvature of 0.1 mm^−1^. With this integration scheme, the FBG unit fits more securely to the glove than the direct fit and is less likely to be pulled, twisted, or deformed during use. This enhances the accuracy of hand posture monitoring, interface consistency, and durability. Furthermore, the stitching points are situated outside the sensing unit, permitting the sensing unit to adjust according to finger movements to capture subtle movements more effectively.

When the sensor records the maximum joint rotation angle (100°), the Bragg wavelength of each cell drifts by around 700 pm. The impact of temperature causes the Bragg wavelength of each FBG to drift by the same quantity, and the temperature sensitivity of the FBGs is only around 6 pm/°C. As the experiments are performed at room temperature, the effect of temperature on the joint rotation angle measurements can be disregarded. For individual fingers, serial arrays are employed, comprising FBGs centered around 1550 nm, 1558 nm, and 1566 nm. These are used to measure MCP, PIP, and DIP joints, respectively. This choice is made to guarantee that the data acquisition of the other sensing units will not be affected by damage to the sensing unit or outliers, and that only one FBG would require replacement.

The system is illustrated in Figure 2b, comprising five parallel FBG arrays connected to the five channels of the FBG demodulator. A demodulator system using LabVIEW is designed, which records data at 1 kHz frequency. When the finger is flexed, the Bragg wavelength drifts with the rise in the axial strain. The host computer captures the drift value and converts it into an angular change value using a mathematical model, which is then transmitted to the hand model. The reconstructed model recognizes the gesture and retrieves it through the embedded SVM program based on the constraints and real-time demodulation data for object prediction.

## 3. Results and Discussions

### 3.1. Measurement Range and Accuracy Test

The Bragg wavelength drift and angle of the rotation data recorded by the IMU sensor were analyzed using the following procedure to evaluate the accuracy and repeatability of the glove’s joint measurements.

1. Mounting the IMU sensor: fasten the IMU sensor onto the hand wearing the FBG sensor, ensuring it is secure and in a steady position.
2. Hand calibration: the hand is placed flat on a level table, whereby the Bragg wavelength of each FBG unit is recorded as a distinctive mapping reference at 0°.
3. Bend the finger joints: bend the finger joints at approximately 1°/s and gradually increase the joint angle from 0°.
4. Data acquisition: commence data acquisition with the self-made data acquisition software, compare the Bragg wavelength value of each FBG unit acquired in real-time with the mapped value at 0°, and calculate the Bragg wavelength drift value at that point. Real-time angle values are obtained from the regression model and are reconstructed for the hand pose in real-time.
5. Real-time angle values are acquired via regression modeling and the real-time reconstruction of the hand posture along with the recognition of the current gesture.
6. Using the recorded values of each joint during object grasping as the training set, the angular values of each joint are computed in real-time. These computed values serve as test set inputs, which are then passed to the embedded MATLAB SVM model in LabVIEW for hyperplane delineation. The model predicts the grasped object accurately.

The aforementioned steps enable the establishment of a correlation between the Bragg wavelength fluctuation of the FBG sensor and the joint angle detected by the IMU sensor. This correlation can be utilized to conduct precision and consistency assessments, which evaluate the sensor’s ability to measure hand positioning. It is essential to ensure the stability of the experimental environment and sufficient data sampling for each joint angle position during data acquisition and mapping relationship construction to obtain dependable and precise outcomes.

**Figure 3:**
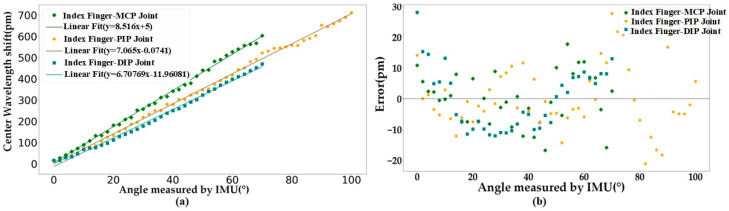
(**a**) Sensitivities of the sensing units and (**b**) the corresponding residual error plot for the index finger.

The results of the calibration are presented in Figure 3a, demonstrating a significant linear correlation between the amount of the Bragg wavelength drift of the MCP and PIP joints, as well as the DIP joints of the index finger (IF), and their joint angle variations. The correlation coefficients stand at 0.995, 0.997, and 0.998 with high clarity, and the findings highlight the association between the two variables. The MCP and PIP joints have a measurement range of 0–70° and 0–100°, respectively. These ranges are wider than those reported in previous studies (60° and 80° in Ref. [16]), allowing a greater diversity of movements to be captured. Furthermore, the DIP joint is independently measured with a range of 0–70°, which is more suitable for analyzing finger dexterity movements than traditional kinematic derivation methods. The sensor demonstrates sensitivities of 8.516 pm/° for the MCP joint, 7.065 pm/° for the PIP joint, and 6.708 pm/° for the DIP joint of the IF, all of which are higher than the sensitivity values reported in reference [20] (5.6 pm/°), and the average increase in sensitivity for the same joints is about 1.4 pm/°. This demonstrates that the designed sensing glove has higher sensitivity in measuring and detecting changes in the angle of the finger joints. The residual plots are displayed in Figure 3b. The regression’s normalized residuals are distributed uniformly on both sides of the Y = 0 line and within the Y = ± 30 pm range. This pattern suggests that the data conform to the requirements of linear regression and exhibit a high degree of linearity. Hence, to ensure computational efficiency for attitude reconstruction, we have opted for a linear regression model (y = ax + b) to transform wavelength drift values into model angles.

### 3.2. Repeatability and Consistency Testing

**Figure 4:**
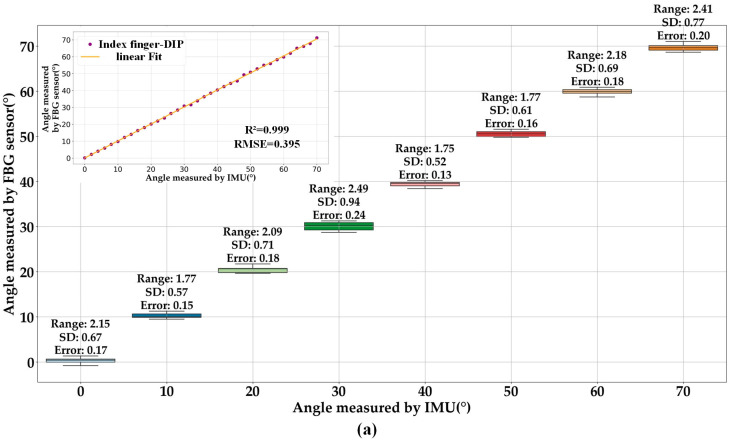

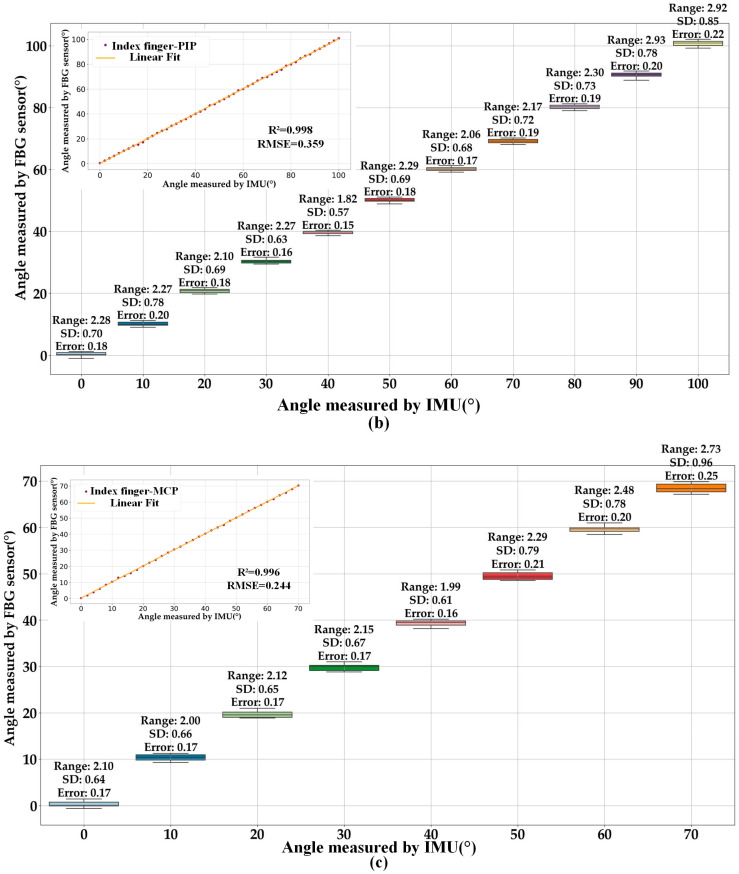
Repeatability test, (**a**) DIP, (**b**) PIP, (**c**) MCP of index finger (inset demonstrates a comparison of the agreement between the angle measurements obtained through FBG and IMU during a single trial).

To assess the repeatability and consistency of the sensing unit measurements, we flex each joint of the finger in increments of 10° and perform five repetitions of flexion-extension for each angle. We analyze the obtained data by applying a linear regression model and generating box-and-line plots, as shown in Figure 4a–c, to assess the repeatability of the measurement, and these box-and-line plots represent the DIP, PIP, and MCP joints of the IF, respectively. Subsequently, we compare the angle measured by the FBG sensor that we created with the true angle measured by the IMU. To better understand the variability in the measured data, the repeated measurements for each angle undergo statistical analysis. This involves calculating the range, SD, and Error value of the repeated measurements data for each angle. This helps provide a comprehensive approach to assessing measurement repeatability and consistency. Table 1 presents a detailed overview of the statistical results.

During the experiment, the angles of the MCP, PIP, and DIP joints are measured, and the different angles of the same joint are analyzed for multiple measurements. The detailed results of one experiment for the DIP, PIP, and MCP joints of the IF are presented in Figure 4a–c, respectively. It is evident from the boxplots that the PIP joint shows the widest range of angular fluctuation at 90° repeated bending, reaching 2.93°, amongst the IF joints. Additionally, the MCP joint exhibits an SD of 0.96° at 70° repeated bending. Furthermore, the MCP joint has an error of 0.25° at 70° repeated bending. Notably, the measurements do not contain any outliers. Due to its smaller fluctuation amplitude, highly focused measurements, and low error rate, it can be concluded that the FBG sensing unit demonstrates good stability and accuracy in angular measurement. Subsequently, the angle data obtained through the IMU are compared and analyzed against the angle data recorded by the FBG sensor. The illustrations in Figure 4 depict a detailed comparison between the angle data obtained from the FBG and IMU measurements from one experiment. The R^2^ values show a significant correlation between the two sets of data with a range of 0.996 to 0.999. It is worth noting that the gradient between the data is near 1.

For the 14 hand joints, Table 1 shows that the MCP joint of the thumb has the highest range value at 3.145° and the highest SD value at 0.941°. Additionally, the PIP joint of the thumb still has the highest Error value at 0.264°. The complex physiology and movement of the thumb are the main reason for this distribution of Error. From the experimental results, it appears that the SD of the MCP joints is larger than that of the PIP joints for each finger except the thumb, which is consistent with the previous literature [23]. This protocol exhibits a minimum error of only 0.176°, which is lower than a similar test protocol [16] that records a minimum error of 0.49°. The mean SD of the protocol is 0.796°, while the mean range is 2.585°, indicating that the test results possess excellent reproducibility with a low value.

In summary, the results indicate a significant agreement between the angle data obtained from the measurements of FBG sensors and IMU, as well as the high repeatability of the FBG sensors in this configuration. This lays a solid foundation for subsequent analytical studies on gesture recognition and the prediction of object grasping.

The average indicates the average of multiple measurements of the same joint at different angles.

### 3.3. Gesture Recognition

**Figure 5:**
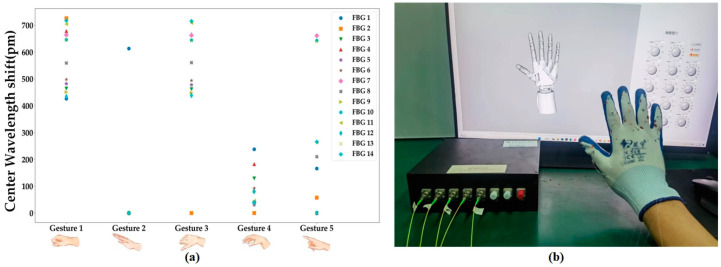
(**a**) Wavelength drifts for Corwin’s gesture, (**b**) the benchmark gesture.

**Figure 6:**
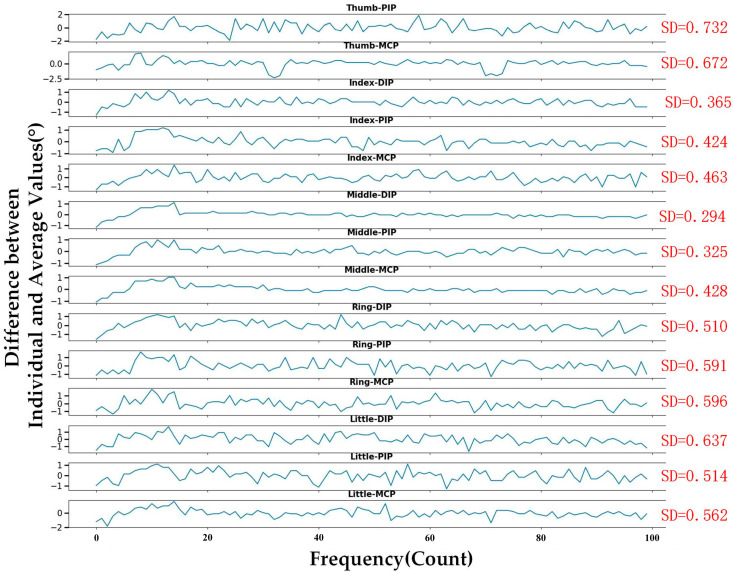
Stability for static gesture (Gesture 1).

To assess the efficacy and dependability of FBG gloves in recognizing gestures, a study is performed to test the recognition ability of five hand gestures. Initially, we establish a hand motion capture system utilizing a 3D virtual model capable of precisely recording the current gesture. The model construction process involves the following steps: firstly, build the individual parts (comprising joints, linkages, and actuators) based on the dimensions of the original manipulator; secondly, assemble these parts to match the topology of the human hand; and lastly, apply rotational constraints on the joints to limit the model’s range and direction of motion, so as to ensure full compatibility with human kinematics. When conducting the Colvin gesture recognition experiments (excluding repetitive gestures), the average of the Bragg wavelength displacement RMS values are recorded for each joint for each of the five gestures. These results are depicted in Figure 5a, while the calibrated gestures from the experiments are illustrated in Figure 5b. Subsequently, the Colvin gesture data are analyzed and the results are presented in Table 2. From the data, it is evident that the highest SD value arises at the MF-MCP joint, reaching 1.42°. When compared to the study in reference [7] that used forearm muscles for gesture recognition, and achieved a maximum SD of 2.19°, the current glove design displays more consistent performance in recognizing gestures. Figure 6 further displays the data obtained when Gesture 1 is performed. After calculating the average values for each joint, we determine the difference between each individual value and its mean value. The reduced SD values indicate that the monitoring angle remains relatively stable while performing the gesture. In summary, the FBG glove currently designed can acquire real-time gesture information with accuracy during gesture capture. It also offers reliable gesture recognition performance. Real-time gesture recognition is detailed in the Supporting Material.

### 3.4. Grabbing Object Recognition

**Figure 7:**
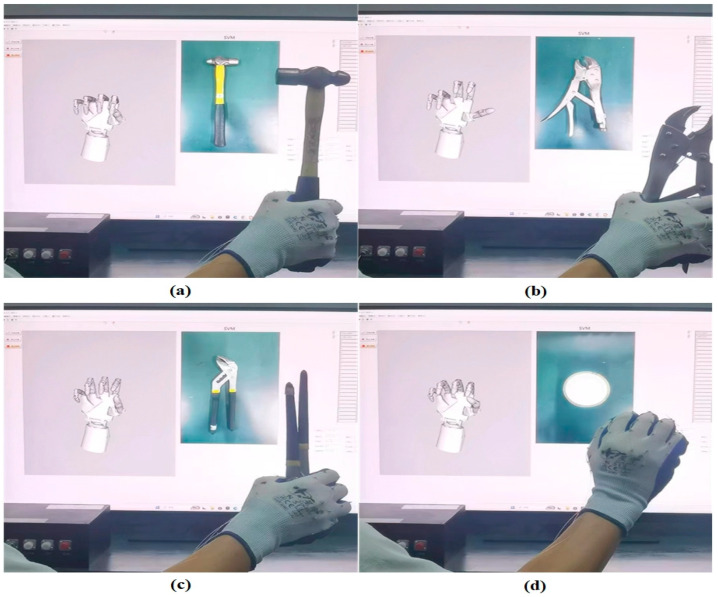
Prediction of grasping objects using SVM (Support Vector Machine), (**a**) industrial hammer, (**b**) vise, (**c**) claw hammer, and (**d**) industrial disk.

To enhance the practicality of the FBG glove within the realm of engineering, we employ the integration of MATLAB into LabVIEW to forecast the manipulation of objects. We use a combination of the angles of the individual joints recorded while grasping different objects as training data for the SVM network, which uses a radial basis function (RBF) for hyperplane segmentation. During the testing phase, the system acquires real-time angle data from the FBG demodulator, which is linearly transformed to attain angular information connected with the wavelength offset and utilized for hyperplane delineation. The main components of the system code consist of data pre-processing, SVM network training and testing, and the post-processing of results. Initially, MATLAB performs data pre-processing, which involves normalizing the samples and their corresponding labels in the training and testing sets and mapping them to the range [0, 1] for consistent data handling. After that, SVM training and prediction take place. Using the standardized training data, we utilize the RBF kernel function as the kernel function of the SVM model that is trained according to hyperplane segmentation. This enables us to construct a network model that can discern the shape of hand gestures. During the testing phase, we apply the trained SVM model to forecast the real-time multi-joint angle data set, thereby identifying the shape details of the predicted object via the use of hyperplane division. Finally, we perform inverse normalization on the results, convert the predictions into real objects, and showcase them through LabVIEW upscaling. The experiment’s prediction concerning object grasping is illustrated in Figure 7. Grasping object predictions are detailed in the Supporting Material S2.

## 4. Conclusions

In this investigation, we propose a hand posture sensor using an FBG array to monitor hand posture in real-time. PDMS with silicone tubing serves as the encapsulation structure for the sensors. The eccentric structure enhances sensitivity and extends the measuring range by arranging the sensors in a sinusoidal shape. In addition, a virtual reality platform utilizing the FBG glove is created in conjunction with LabVIEW and MATLAB to showcase the feasibility in real-world applications. The experimental results demonstrate that the FBG glove proposed here has greater accuracy (Error = 0.176°), enhanced stability (SD = 0.685°), and the ability to measure the movement of fourteen finger joints in real-time, as compared to the previously suggested FBG glove system. Currently, the primary limitation of the glove lies in its use of PDMS flexible encapsulation material, which exhibits a strain hysteresis effect. This phenomenon causes a relatively obvious delay in the response when subjected to a large strain, thereby potentially impacting the real-time performance of the glove. Such an effect would be particularly problematic when swift and precise gesture recognition or motion tracking is required. To overcome this limitation, we are currently exploring techniques to enhance the materials and optimize algorithms, thereby improving the performance and minimizing the impact of delayed responses on the system. These improvements will serve as the foundation for future human–computer interaction applications and digital twin applications. In conclusion, the proposed FBG posture sensor possesses the benefits of being simple, repeatable, and having a fast response and stability. Applying the hand posture sensors to each joint enables us to monitor the abduction and adduction movements of all joints in real-time, as well as the hand posture and prediction of the shape of the grasped object.

## Figures and Tables

**Table 1:** Repetitive flexion and extension test analysis.

Repeatability Index	Average Range (°)	Average SD (°)	Average Error (°)
TF-PIP	2.320	0.831	0.264
TF-MCP	3.145	0.941	0.243
IF-DIP	2.076	0.685	0.176
IF-PIP	2.310	0.711	0.184
IF-MCP	2.233	0.720	0.188
MF-DIP	2.480	0.761	0.198
MF-PIP	2.825	0.836	0.217
MF-MCP	2.628	0.848	0.220
RF-DIP	2.585	0.784	0.204
RF-PIP	2.785	0.825	0.215
RF-MCP	2.701	0.841	0.218
LF-DIP	2.533	0.774	0.200
LF-PIP	2.514	0.768	0.198
LF-MCP	2.768	0.814	0.209
Mean value	2.585	0.796	0.210

Thumb finger (TF), middle finger (MF), ring finger (RF), little finger (LF).

**Table 2:** Colvin gesture data analysis.

Stability (SD)	Gesture 1	Gesture 2	Gesture 3	Gesture 4	Gesture 5
TF-PIP	0.732	0.685	0.688	0.863	0.690
TF-MCP	0.672	0.807	0.750	0.687	0.832
IF-DIP	0.361	0.347	0.759	0.715	0.379
IF-PIP	0.424	0.340	0.782	0.552	0.555
IF-MCP	0.463	0.453	1.16	0.741	0.789
MF-DIP	0.294	0.898	0.608	0.560	0.550
MF-PIP	0.325	1.07	0.783	0.585	0.615
MF-MCP	0.428	1.42	0.864	0.844	0.744
RF-DIP	0.510	0.720	0.967	0.586	0.473
RF-PIP	0.591	1.08	0.699	0.632	0.628
RF-MCP	0.596	0.968	0.923	0.569	0.542
LF-DIP	0.637	0.901	0.692	0.676	0.600
LF-PIP	0.514	0.839	0.847	0.527	0.757
LF-MCP	0.562	1.08	1.02	0.556	0.777

SD is used to indicate the stability of the data collected by each sensor when making a gesture.

## Data Availability

Not applicable.

## Supplementary Materials

The following supporting information can be downloaded at: https://www.mdpi.com/article/10.3390/s23208495/s1, Video S1: Real-time gesture recognition, Video S2: Grasping object prediction (SVM).
